# Roles of acidic residues in SpeG acetyltransferases—insights into importance for kinetic activity and polyamine binding in allosteric and acceptor sites

**DOI:** 10.1042/BCJ20260162

**Published:** 2026-06-26

**Authors:** Hazel N. Leiva Martel, Van Thi Bich Le, Ekaterina V. Filippova, Aron W. Fenton, Melissa Law, Martha Marquez-Ramirez, Misty L. Kuhn

**Affiliations:** 1 Department of Chemistry & Biochemistry, San Francisco State University, San Francisco, California, U.S.A.; 2 Department of Biochemistry and Molecular Biology, Institute for Biophysical Dynamics, University of Chicago, Chicago, Illinois, U.S.A.; 3 Department of Biochemistry and Molecular Biology, The University of Kansas Medical Center, Kansas City, Kansas, U.S.A.

**Keywords:** allosteric enzyme, Gcn5-related N-acetyltransferase (GNAT), homotropic and heterotropic allosteric enzyme, polyamines, spermidine/spermine N-acetyltransferase (SSAT), substrate specificity

## Abstract

The Gcn5-related *N*-acetyltransferases belong to a massive superfamily of enzymes that perform a wide array of functions in different organisms. This family is comprised of smaller subfamilies, including one called the spermidine/spermine *N*-acetyltransferases (SSATs). SSATs acetylate positively charged long-chain polyamines to maintain their intracellular concentrations. In bacteria, one primary type of SSAT is the SpeG enzyme, which adopts a homododecameric assembly. In the present study, we sought to detail how polyamines bind to both the allosteric and active sites of SpeG and determine which conserved acidic and polar residues are critical for kinetic activity and polyamine binding. Therefore, we determined a crystal structure of the *Vibrio cholerae* (VcSpeG) enzyme in complex with spermine in the allosteric site and *N*^1^-acetylspermine in the active site. This result clearly defines two distinct and separate polyamine binding sites within the protein. Furthermore, it demonstrates that SpeG is indeed an allosteric enzyme: homotropic in that the ligands are identical and heterotropic in that the allosteric binding sites are distinct from the active sites. We also investigated the kinetic activity of substituted residues in both sites and found several residues are critical for enzyme activity, while some substitutions altered polyamine substrate specificity. These combined structural and functional results begin to illuminate how longer-chain polyamines with terminal aminopropyl groups are recognized and acetylated by SpeG. Finally, we present a hypothetical model for proposed roles of conserved acidic residues in both sites, which provides a framework for subsequent studies of SpeG’s intricate allosteric network.

## Introduction

Gcn5-related *N-*acetyltransferase (GNAT) enzymes are widespread throughout all facets of life and are some of the most sequence- and structurally-diverse among all known enzymes. GNATs have evolved to accommodate a dearth of chemically variable substrates, and they exhibit different types of oligomeric assemblies. Despite sharing a common overall structural fold, our understanding of how substrates are recognized and utilized by each type of GNAT enzyme remains limited. In general, GNATs catalyze a bisubstrate reaction where a donor molecule, such as acetyl coenzyme A (AcCoA), donates its acyl group to the primary amine of an acyl acceptor substrate molecule. Acceptor substrates range in size from small molecules to larger macromolecules, and thus the acceptor sites of GNAT enzymes have evolved to accommodate substantially chemically diverse substrates. One limiting factor in understanding how these enzymes discriminate between substrates and which residues are important for kinetic activity is the relatively low number of protein structures that have been determined in complex with acceptor substrates. Therefore, an overarching goal of our laboratory has been to expand the number of GNAT protein crystal structures with ligands bound and use these structures and enzyme kinetics to identify which residues are critical for enzyme activity and ligand specificity.

Spermidine/spermine *N-*acetyltransferases (SSATs) belong to one subfamily of GNAT enzymes that presents a unique opportunity to explore their novel structural and functional properties. SSATs are critical in bacteria for maintaining a proper balance of intracellular concentrations of polyamines. These small molecules carry a positive charge at physiological pH, which serves as counterions and aids many important biological processes [1–3]. The role of SSAT enzymes is to partially neutralize the positive charge of polyamines *via* acetylation, thereby allowing these molecules to be exported and helping to maintain intracellular pH. This is critical because in some bacteria high concentrations of polyamines lead to cellular toxicity and decreased viability [4–6]. Polyamines can also mediate the pathogenicity of bacteria in multiple ways, including making them more susceptible to antibiotics [7], affecting virulence [8], and altering biofilms in some organisms [9,10]. Therefore, understanding the molecular details of how SSAT enzymes regulate polyamine concentrations has promise for developing novel enzyme therapeutics to treat bacterial infections.

Interestingly, bacterial SSAT enzymes are known to adopt unique quaternary structural arrangements, regulatory properties, and ligand binding compared with their eukaryotic homologs. One example of this variability includes the bacterial SpeG SSAT enzyme, which adopts a higher-ordered dodecameric oligomeric state [11–13]. Most other SSATs are monomers or dimers [14–16]. To date, SpeG enzymes are the only type of GNAT that have been described to exhibit dodecameric protein assembly. In SpeG enzymes, the higher-ordered oligomeric state enables significant interfacial contacts between protomers, helps to stabilize a mobile allosteric effector loop and bind allosteric effectors, and enables longer effector molecules to not only bind within the allosteric site but also to adjacent protomers, which tightens the dodecamer and alters the overall electrostatic surface [11,13,17,18]. The allosteric sites are not present in eukaryotic homologs since their quaternary structures are C-terminal domain-swapped dimers that do not form higher-ordered oligomers [19]. Our previous studies have shown that the allosteric site binds either spermine (Spm) or spermidine (Spd) as effectors, and it is located approximately 15 Å from the active site when measuring the distance between the catalytic tyrosine residue in the active site and the closest glutamate residue in the allosteric site (Y134 and E33, respectively, in *Vibrio cholerae* SpeG) [11]. We have also shown that SpeG and other SSATs acetylate Spm and Spd [11,20–22], and under certain assay conditions both polyamines are acetylated with nearly identical catalytic efficiencies (*k*_cat_/*K*_m_ or *k*_cat_/*S*_0.5_) [17,23]. This acetylation occurs within the active site that is divided into two ligand-binding sites created by a beta-bulge within the central beta-sheet of the enzyme: the acyl-donor site where AcCoA binds and the acyl-acceptor site where polyamine binds. While the AcCoA binding location is conserved across many SSAT enzymes, the polyamine substrate binding site varies, and there is currently no consensus regarding which residues are required for polyamine substrate specificity across different types of SSATs [11,14,19,24,25]. This exemplifies a significant knowledge gap regarding how different polyamine molecules are selected for and bind to acceptor sites of SpeG SSAT enzymes.

SpeG enzymes are also rather unique among well-described allosteric enzymes because their allosteric effector and acceptor substrate are identical polyamine molecules (i.e*.*, a homotropic allosteric enzyme with respect to the identity of the ligands). Given that the allosteric site is formed at a distinct site of the protein chain relative to the active site, this system is also considered a heterotropic allosteric system with respect to the ligand-binding sites. The allosteric sites of SpeG proteins are created when a mobile loop (residues 22–37 in *Vibrio cholerae* SpeG) near the N-terminus of the protein transitions to a more ordered alpha helix, which allows polyamines to bind at this site [11,17,18,26]. This conformational change and subsequent binding of polyamine causes adjacent protomers to form tighter interprotomer interactions [12,13,17]. Smaller polyamines like Spd bind within a single protomer allosteric site, and longer polyamines, such as Spm, form additional interactions with adjacent protomers beyond the allosteric site [11–13,17,26,27]. While many of the prior SpeG crystal structures we determined in complex with polyamines in allosteric sites have revealed residues that are important for coordinating polyamines in these sites, a knowledge gap still remains regarding the importance of these residues on polyamine binding, specificity, and turnover.

Since SpeG enzymes regulate intracellular polyamine concentrations, and many bacteria rely on polyamines for biofilms and other cellular processes, a fundamental understanding of polyamine selectivity in these enzymes is important. Therefore, we chose the SpeG enzyme from *Vibrio cholerae* (VcSpeG) for the present study because this pathogen has an intricate and highly regulated polyamine signaling system that enhances or disperses biofilms [28–30]. Additionally, our prior studies with this enzyme have shown that it can be heterologously expressed in large quantities in *Escherichia coli* and is amenable to crystallization. We have crystallized VcSpeG in the presence of multiple physiologically relevant ligands and in a variety of conformational states [11,13]. Additionally, we have shown that VcSpeG exists in a dynamic equilibrium between different oligomers (dimer, tetramer, and dodecamer) depending upon protein concentration and whether polyamine is present [12,13]. Despite having significant structural data for this enzyme, only the location of the polyamine in the allosteric site has been determined, and we do not have a solid understanding of which residues in the acceptor site are critical for polyamine binding and turnover. Thus, the aim of the present study was to obtain a crystal structure of the VcSpeG protein in complex with polyamine in both its allosteric and acceptor sites and examine the importance of key residues within both of these sites on the enzyme’s function.

## Results

### Two distinct polyamine binding sites are present in SpeG proteins: the allosteric site and the acceptor site

**Figure 1:**
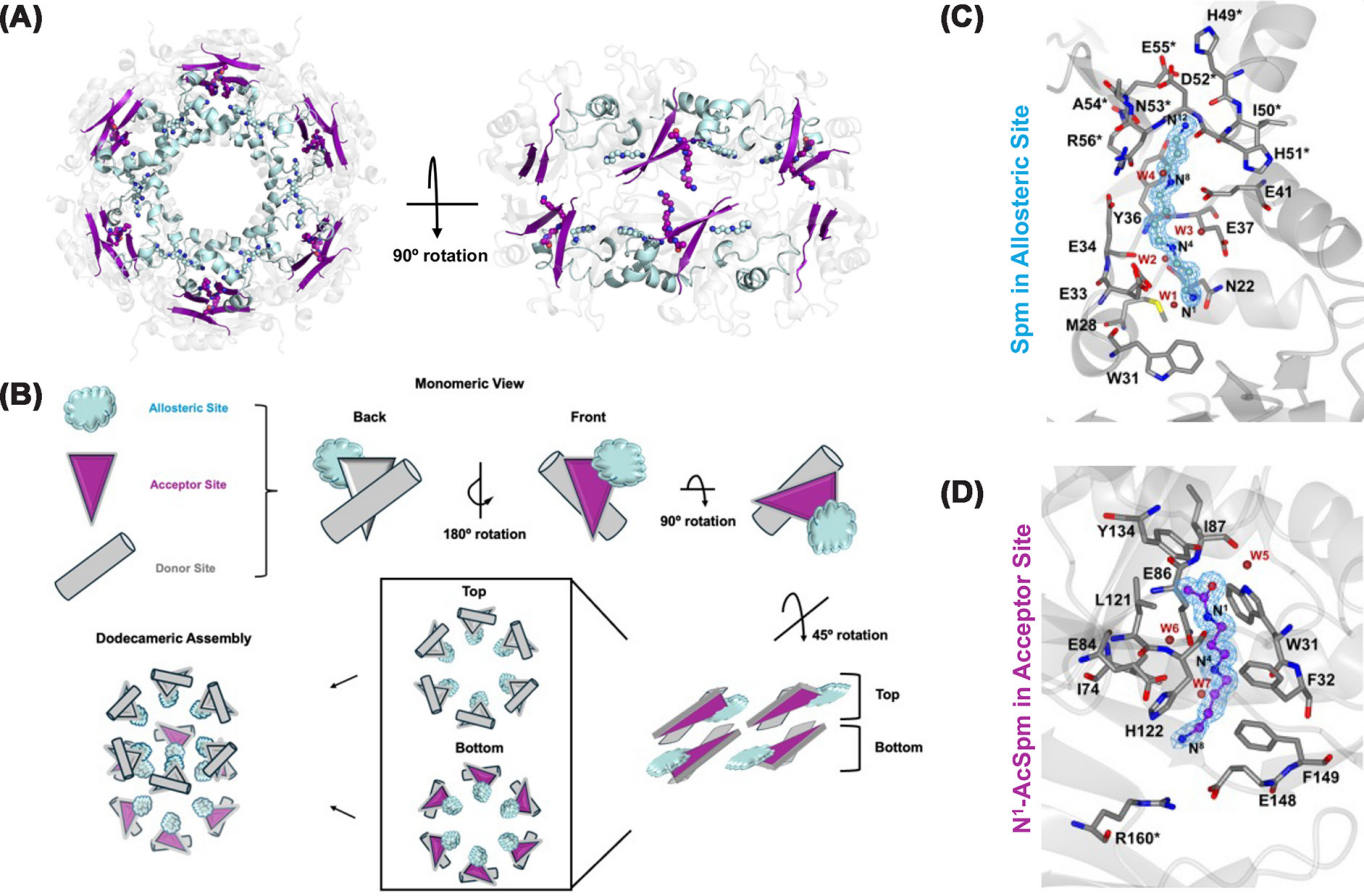
Crystal structure of WT VcSpeG protein in complex with Spm and AcSpm at 1.35 Å resolution and arrangement of protomers in the dodecamer (**A**) Top and side views of the dodecameric assembly of the VcSpeG 6E1X structure in complex with *N*^1^-AcSpm in the acceptor site (purple spheres) and Spm in the allosteric site (light blue spheres). The secondary structures of the allosteric site are colored in light blue, and the V-splay that creates the active site of the protein is colored in purple. (**B**) Cartoon representation of the location of the donor (gray cylinder and backside of the triangle), acceptor (purple triangle), and allosteric sites (blue cloud) on a single protomer compared with dodecamer assembly. The top and bottom hexamers stack on top of each other in an orientation that enables the acceptor sites to be facing inward. (**C,D**) Electron density omit maps surrounding ligands (Spm: top, light blue; *N*1-AcSpm: bottom, purple) in chain C of the WT VcSpeG crystal structure. Protein residues are shown in gray with non-carbon atoms colored in blue for nitrogen and red for oxygen. Water molecules are shown with brown spheres and labeled W1–W4. Note: The nitrogen atoms for Spm in the crystal structure are denoted as *N*^1^, *N*^5^, *N*^10^, and *N*^14^, but the convention for referring to nitrogens within polyamines, and the Spm molecule specifically, is *N*^1^, *N*^4^, *N*^8^, and *N*^12^. Throughout the article, we use the latter conventional nitrogen numbering.

To gain a greater understanding of how polyamines bind to the acceptor portion of the active site of SpeG enzymes, we co-crystallized the VcSpeG protein with *N*^1^-acetylspermine (*N*^1^-AcSpm). The protein crystallized as a well-ordered dodecamer at 1.35 Å resolution, with six protomers present in the asymmetric unit (PDB ID: 6E1X; Table 1 and Figure 1). Within the crystal lattice, these six protomers assemble into a dodecamer through a crystallographic two-fold rotation axis, which is consistent with our prior studies with this enzyme [11]. Surprisingly, we observed non-acetylated Spm within all allosteric sites of the protein (chains A–F; Figure 1 and Supplementary Figure S1) despite not adding this compound to co-crystallization solutions. We suspect this contaminant Spm was present in the commercially supplied *N*^1^-AcSpm. Electron density clearly surrounds the entire non-acetylated Spm ligand in the allosteric site in four protomers (chains A–D), whereas partial disorder of the *N*^1^-aminopropyl group was observed in two protomers (chains E and F) (Figure 1C and Supplementary Figure S1). This observed partial disorder of the ligand in chains E and F may be due to the limited concentration of contaminant Spm present in solution. Importantly, no additional density that would correspond to an acetyl group of *N*^1^-AcSpm was detected, which indicates that the ligands in these allosteric sites are more consistent with Spm rather than *N*^1^-AcSpm. On the other hand, we observed *N*^1^-AcSpm within the acceptor site of four protomers (chains A–D; Figure 1D and Supplementary Figure S2): The *N*^1^-acetylated end of *N*^1^-AcSpm, as well as the aminopropyl and aminobutyl moieties that include *N*^4^ and *N*^8^, were well-ordered and formed significant interactions with residues in the acceptor site (described below) (Figure 1D and Supplementary Figure S2). The *N*^12^-nonacetylated aminopropyl end of the molecule was more flexible and exhibited reduced binding, as indicated by partial occupancies and disorder in some protomers (Supplementary Figure S2). Additional ligands from crystallization, including MPD/MRD ((4S/4R)-2-methyl-2,4-pentanediol) and Tris buffer, were present in the structure, but they did not appear to compete with polyamine binding at either allosteric or acceptor site (Supplementary Figure S3). Thus, this structure confirms that SpeG proteins are a unique example of homotropic allosteric enzymes with respect to the ligand identity and heterotropic allosteric enzymes whereby two separate polyamine binding sites are present: the allosteric site where non-acetylated Spm binds and a separate acceptor site where Spm is acetylated to form *N*^1^-AcSpm product.

**Table 1:** X-ray data collection and refinement statistics of two SpeG structures from *Vibrio cholerae.* Highest resolution shell statistics are shown in parentheses

**PDB ID**	**6E1X**	**6DAU**
	WT in product-bound state	E33QE41Q substitution
**Crystal parameters:**		
Resolution (Å)	30.0–1.35 (1.37–1.35)	30.0–2.26 (2.3–2.26)
Space group	C222	C121
Unit cell parameters		
a, b, c (Å)	185.98, 186.5, 73.72	160.23, 133.02, 77.71
α, β, γ (°)	90, 90, 90	90, 113.39, 90
Matthews coefficient (Å3/Da)	2.54	3.06
Solvent content (%)	51.6	59.8
**Data collection:**		
Completeness (%)	99 (97.8)	98.7 (100)
No. of unique reflections	276661	68104
I/σ(I)	33.9 (3.1)	30.2 (1.93)
R_merge_ (%)	0.05 (0.65)	0.06 (0.64)
CC1/2	(0.851)	(0.751)
Redundancy	7.5 (7.4)	3.7 (3.7)
Wilson B-factor (Å^2^)	13.9	62.5
**Refinement:**		
R (%)/R_free_ (%)	12.3/15.1	18.9/21.6
r.m.s.d. bond length (Å)	0.015	0.013
r.m.s.d. bond angle (°)	1.7	1.6
Average *B* value (Å^2^)	18.6	74.1
No. of molecules in AU	6	6
No. of atoms		
Protein	8681	8569
Water molecules	1469	217
**Ramachandran analysis:**		
Favoured (%)/n	100/1097	99.0/997
Allowed (%)/n	-	1/8
Outlier (%)/n	-	-

### Spm and *N*^1^-AcSpm ligands within the allosteric and acceptor sites of VcSpeG, respectively

To detail how polyamines are coordinated within the allosteric and acceptor sites of SpeG, we next examined the residues that interacted with Spm and *N*^1^-AcSpm in these sites in the 6E1X crystal structure.

#### Spm in the allosteric site

A combination of side chain, backbone, or water-mediated interactions with each nitrogen atom (*N*^1^, *N*^4^, *N*^8^, and *N*^12^) of the Spm ligand was observed in the allosteric site (Figure 1C). For our analysis, we focused on the allosteric site located within chain C and its interactions with chain B because it contained the most well-ordered Spm ligand among all six protomers. Within this binding site, the E33 residue adopted alternative rotamer conformations as indicated by partial electron densities, with one conformation forming a direct interaction between the E33 OE1 side chain and the *N*^1^ atom of Spm and another conformation forming a water-mediated hydrogen bond between the *N*^1^ atom of Spm and water molecule W1. Additional water-mediated hydrogen bonds were observed with the backbone oxygen atom of E34 and the *N*^4^ atom of Spm through W2; the E34 side chain formed a salt bridge with R56 of the adjacent protomer (chain B). E37 and E41 formed water-mediated side chain interactions with the *N*^4^ atom of Spm through hydrogen bonds with W3. The OE2 atom of E41 interacts directly with the *N*^8^ atom of Spm. Additional water-mediated interactions between the ND2 atom of the N53 side chain of the adjacent protomer (chain B) and the *N*^8^ atom of Spm occur through hydrogen bonds with W4. The adjacent protomer interacts with the *N*^12^ atom of Spm through backbone interactions with H49, I50, and D52. In two protomers of the complex structure (chains E and F), we observed incomplete binding of Spm in these allosteric sites. This appears to correlate with an alternative conformation of the allosteric loop and conformational heterogeneity of two key aromatic residues on the loop: W31 and Y36 (Supplementary Figure S4).

When we compared these interactions (PDB ID: 6E1X) with our previous crystal structures of VcSpeG in complex with Spm (PDB ID: 4MI4) or Spd (PDB ID: 4MHD) in the allosteric site, we found the majority of the interactions between side chains and ligands in this site were consistent across crystal structures. The only deviations were in the way the *N*^1^ and *N*^4^ amines of Spd interacted due to its shorter length of one aminopropyl group compared with Spm. In the 4MHD structure, the *N*^1^ terminal amine of Spd interacts directly with the side chains of E33 and E37 (chain B), and the *N*^4^ amine interacts with the E41 side chain [11]. Thus, the polyamine molecules that bind to the VcSpeG allosteric site are stabilized significantly in this pocket *via* a highly intricate network of acidic residues and water molecules. Additionally, the residues within this site have sufficient plasticity to accommodate and recognize different lengths of polyamines while maintaining key interactions between protomers.

#### *N*^1^-AcSpm in the acceptor site

As noted above, within different protomers of the 6E1X structure, the *N*^1^-AcSpm ligand showed different qualities of electron density that surrounded the non-acetylated end of the molecule. Therefore, we focused on the *N*^1^-AcSpm ligand bound in chain C, since its electron density was the most well-defined. We observed that *N*^1^-AcSpm is anchored in the acceptor site through a series of hydrogen-bonding interactions between water molecules, residues within the acceptor site, and backbone interactions with residues at the interface of the acceptor and donor sites (Figure 1D). Two residues within the acceptor site interacted with *N*^1^-AcSpm directly (Q86) and through water-mediated hydrogen bonds (E84). E84 formed interactions with three of the nitrogen atoms of *N*^1^-AcSpm, including *N*^1^, *N*^4^, and *N*^8^ through water molecules W6 and W7, whereas Q86 interacted directly with *N*^4^ *via* side-chain interactions. The *N*^1^ terminal amine interacted directly with the backbone oxygen atom of H122 and water-mediated interactions *via* backbone oxygen atoms of F85 and nitrogen atom of H122 through W6. The acetyl carbonyl oxygen of *N*^1^-AcSpm formed hydrogen-bonding interactions with the backbone nitrogen atom of I87 and a water-mediated hydrogen-bond through the backbone oxygen atom of I87 through W5. This acetyl group is in a similar location as the acetyl group of the AcCoA substrate in the VcSpeG crystal structure (PDB ID: 4R57) and is in close proximity to the conserved Y134 residue that is known to be critical for activity in the SpeG enzyme from *E. coli*. Thus, the placement of the *N*^1^-AcSpm molecule in the acceptor site is very likely near the position in which Spm becomes acetylated.

Due to the variable quality of electron density around the *N*^12^ terminal amine of *N*^1^-AcSpm in multiple acceptor sites in the 6E1X crystal structure, we note that the terminal end of the molecule is more flexible and therefore hypothesize that recognition of the entire polyamine molecule within the acceptor site may not be required. Instead, the *N*^1^, *N*^4^, and *N*^8^ positions of the polyamine dictate binding and recognition within this site, which explains how longer-chain polyamines with terminal aminopropyl groups and variable structures can bind and become acetylated by the SpeG protein [11,31]. Furthermore, alterations in conformations of the W31 residue, which is positioned between the allosteric and acceptor sites, prevent binding of *N*^1^-AcSpm in two protomers (chains E and F) of the crystal structure (Supplementary Figures S2 and S4). These two protomers also exhibit more disordered electron density around the Spm ligand in the allosteric site. Therefore, the conformations of the residues within the allosteric site and on the allosteric loop are correlated with ligand binding to both allosteric and acceptor sites of the protein.

### Conservation of acidic and polar residues in SpeG allosteric and acceptor sites and selection of residues for site-directed mutagenesis

**Figure 2:**
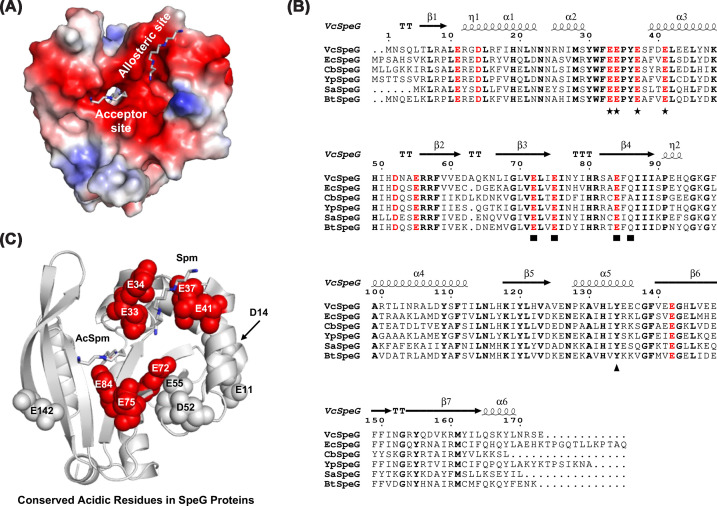
Conservation of acidic residues in SpeG bacterial homologs and their distribution in allosteric and acceptor sites of the VcSpeG protein (**A**) Electrostatic surface of a single VcSpeG protomer (PDB ID: 6E1X chain A) in complex with Spm (allosteric site) and *N*^1^-AcSpm (acceptor site). The surface is colored red (negative charge), blue (positive charge), and white (neutral). Ligands are represented in white sticks. (**B**) Multiple sequence alignments of bacterial SpeG homologs (*Vibrio cholerae* (Vc), *Escherichia coli* (Ec), *Coxiella burnetii* (Cb), *Yersinia pestis* (Yp), *Staphylococcus aureus* (Sa), and *Bacillus thuringiensis* (Bt)) with the secondary structural elements of the PDB ID: 6E1X chain A structure on top of the alignment. Conserved residues are bolded black for non-acidic and red for acidic residues. Residues selected for mutagenesis are shown with black stars (allosteric site), black squares (acceptor site), and a black triangle (donor site) beneath the alignment. Sequences were aligned in Clustal Omega and visualized using ESPript [48]. (**C**) Conserved acidic residues in SpeG bacterial homologs are shown as spheres on a single VcSpeG protomer (PDB ID: 6E1X chain A). Red spheres with white labels correspond to residues that were mutated in the present study. Residues with gray spheres were not selected for mutagenesis but are conserved. Ligands (Spm and *N*^1^-AcSpm) are represented in white sticks.

**Figure 3:**
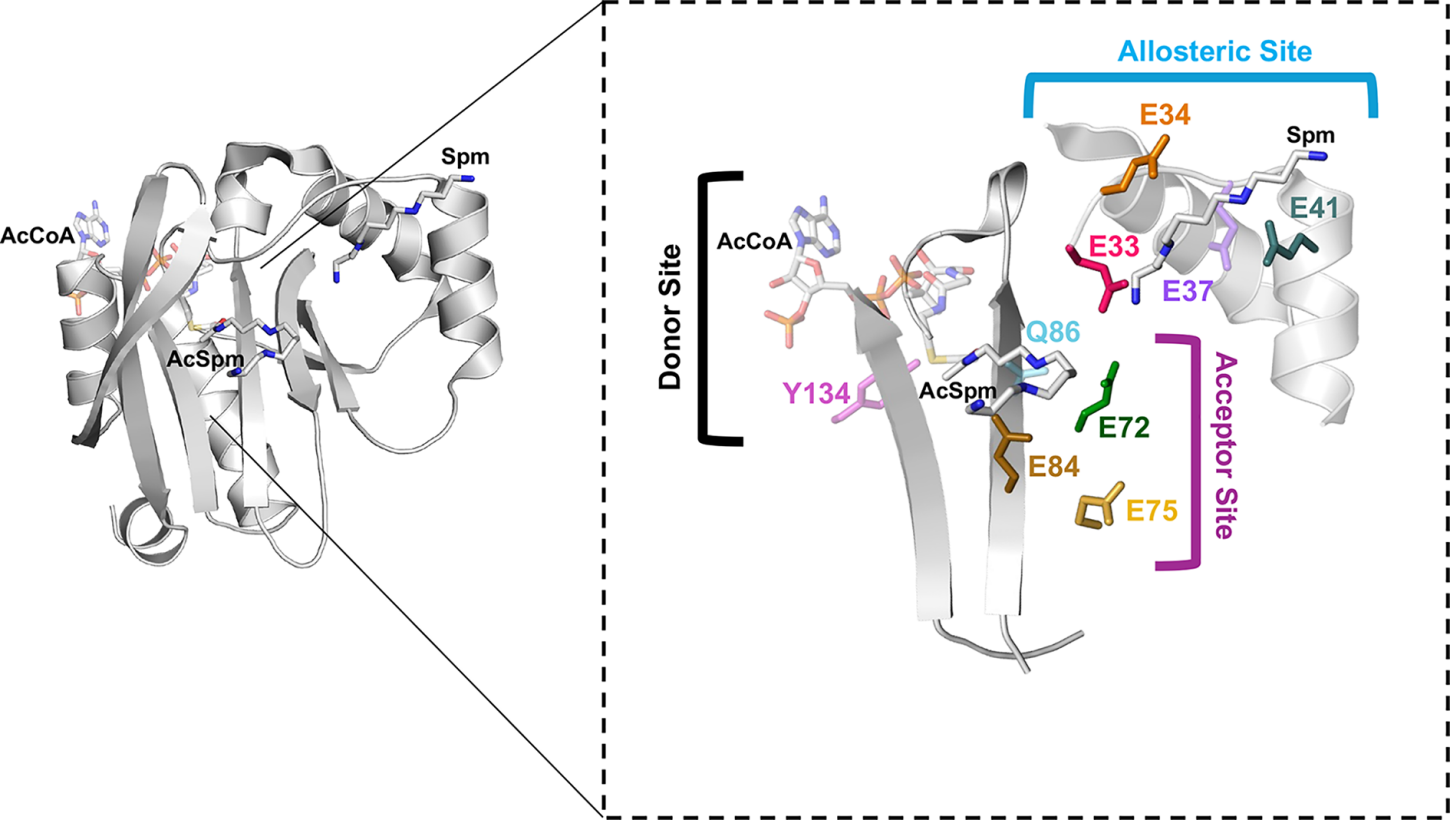
Overview of the allosteric, donor, and acceptor sites in a single protomer of the VcSpeG enzyme A single protomer of the 6E1X crystal structure (chain A) in complex with Spm and *N*^1^-AcSpm is shown with gray ribbons. Spm is located in the allosteric site (blue circle), and *N*^1^-AcSpm is in the acceptor site (purple bracket) and are shown with white sticks. AcCoA was modeled from the VcSpeG 4R57 crystal structure to denote the donor site (gray bracket). Specific residues that were selected for site-directed mutagenesis and enzyme kinetics assays are shown as sticks and differentially colored in the zoomed view. The acceptor substrate site is on the front face of the β-sheet, and the donor site is on the back face of the β-sheet. The figure was made using PyMOL and Microsoft PowerPoint.

After observing that the polyamine ligand interactions in both allosteric and acceptor sites of the VcSpeG protein were dependent mostly on interactions between the amino groups of Spm and acidic residues in both sites, we sought to understand whether these residues were more broadly conserved across other SpeG proteins. Therefore, we examined the conservation of acidic residues within SpeG proteins from other bacterial species that have been structurally characterized and found that ten glutamate and two aspartate residues were conserved (Figure 2). The majority of these conserved acidic residues are within the allosteric (E33, E34, E37, and E41) and acceptor (E72, E75, and E84) sites of SpeG (Figure 2C; red). There are a few additional conserved acidic residues, but those are either surface-exposed or do not appear to contribute to binding allosteric effectors or substrates (Figure 2C; gray); therefore, we did not further consider those other residues. In addition to the acidic residues within the two types of binding sites, we noted that one polar residue (Q86) that directly interacted with *N*^1^-AcSpm in the acceptor site was conserved in all SpeG proteins except CbSpeG (Figure 2). To examine the importance of these eight residues on enzyme function, we substituted all conserved glutamate residues with glutamine to retain polarity but remove the negative charge. This strategy preserves side chain size and ability to form hydrogen bonds, thereby minimizing structural changes within the allosteric site and enabling assessment of the specific roles of electrostatic interactions. In contrast, the Q86 residue was replaced with an alanine to remove the side chain polarity and assess its contribution to local interactions. Finally, Y134 was substituted with alanine since we wanted to determine whether this residue was similarly critical for catalytic activity as we observed previously for the *E. coli* SpeG enzyme [32]. Thus, a total of nine residues were substituted as a means of probing their contributions to kinetic activity in the VcSpeG protein (Figure 3).

### Altering conserved polar residues in the allosteric and acceptor sites exhibit differential effects on kinetic activity toward Spm and Spd

**Figure 4:**
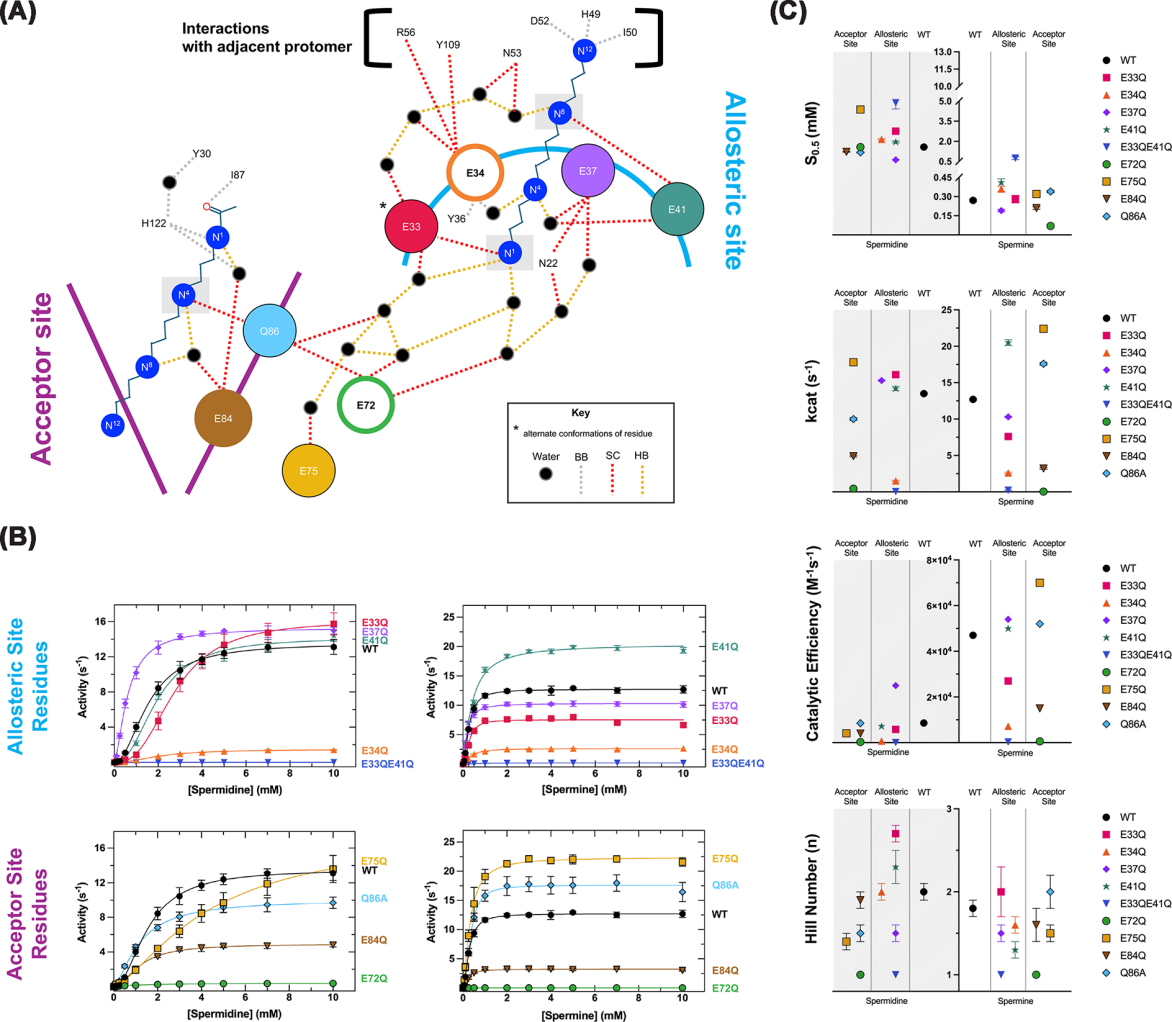
Enzyme activity of VcSpeG WT and substituted proteins and hydrogen-bonding network of WT residues with ligands in the allosteric and acceptor sites of the 6E1X crystal structure (**A**) Cartoon representation of the hydrogen-bonding network between selected acceptor site and allosteric site residues, water, allosteric Spm, and acceptor site *N*^1^-AcSpm of one protomer of the 6E1X crystal structure (chain C). The interactions between two adjacent protomers of a top hexamer of the dodecamer are shown with black brackets. Water molecules are shown as black circles, and backbone (BB) interactions, side chain (SC) interactions, and hydrogen-bonding (HB) interactions are shown as dashed lines that are colored gray, red, and yellow, respectively. Filled circles indicate residues that when mutated minimally decreased catalytic efficiency or improved catalytic efficiency; circles with white interiors indicate residues that were critical for enzymatic activity when substituted. Spm and AcSpm are shown as cartoons with amino groups in blue circles. Gray boxes indicate where VcSpeG residues form side-chain interactions with specific amino groups in the polyamines in chain C of the PDB ID: 6E1X crystal structure. (**B**) Spd and Spm substrate saturation curves for VcSpeG WT and substituted proteins. Labels adjacent to the plot are color-coordinated based on residues selected in Figure 3. (**C**) Visual representation of the kinetic parameters of the WT and mutant proteins toward Spd and Spm. Each parameter is indicated below the *y*-axis and is grouped by WT, allosteric site, or acceptor site mutant and substrate. Numerical data for kinetic parameters are shown in Table 2. The figure was made using GraphPad Prism 10 and Microsoft PowerPoint.

Enzymatic characterization of WT VcSpeG and substituted proteins is included in Figure 4. To facilitate the discussion of results for substitutions made at individual residue positions in the acceptor and allosteric sites, a cartoon diagram is included in Figure 4A. As illustrated in Figure 4B, due to greatly reduced activity, data for E72Q are not easily visible in the same graphs as data for other substitutions and are, therefore, included in Supplementary Figure S5.

#### WT VcSpeG enzyme

In the present study, we showed that the WT VcSpeG enzyme exhibited a higher apparent affinity (*S*_0.5_) (∼6-fold) for Spm compared with Spd, the turnover number (*k*_cat_) was similar for both Spm and Spd substrates, and the enzyme exhibited significant positive cooperativity (i.e., the Hill number; *n*_H_) toward both Spm (*n*_H_ = 1.8) and Spd (*n*_H_ = 2.0) (Table 2), which was consistent with our prior studies [11].

**Table 2:** Kinetic parameters of WT and substituted VcSpeG proteins determined from substrate saturation curves using Spm or Spd as the acceptor substrate

Site	Protein	*S*_0.5_ or *K*_m_ (mM)	*k*_cat_ (s^−1^)	Catalytic efficiency (M^−1^s^−1^)	Hill number (*n*_H_)
	WT Spm	0.27 ± 0.01	12.7 ± 0.1	4.7 × 10^4^	1.8 ± 0.1
	WT Spd	1.57 ± 0.03	13.5 ± 0.1	8.6 × 10^3^	2.0 ± 0.1
Allosteric site residues	E33Q Spm	0.28 ± 0.03	7.6 ± 0.2	2.7 × 10^4^	2.0 ± 0.3
	E33Q Spd	2.76 ± 0.04	16.1 ± 0.2	5.8 × 10^3^	2.7 ± 0.1
	E34Q Spm	0.36 ± 0.02	2.6 ± 0.1	7.2 × 10^3^	1.6 ± 0.1
	E34Q Spd	2.15 ± 0.05	1.5 ± 0.1	7.0 × 10^2^	2.0 ± 0.1
	E37Q Spm	0.19 ± 0.01	10.3 ± 0.1	5.4 × 10^4^	1.5 ± 0.1
	E37Q Spd	0.62 ± 0.02	15.3 ± 0.1	2.5 × 10^4^	1.5 ± 0.1
	E41Q Spm	0.41 ± 0.03	20.5 ± 0.4	5.0 × 10^4^	1.3 ± 0.1
	E41Q Spd	1.96 ± 0.08	14.2 ± 0.3	7.2 × 10^3^	2.3 ± 0.2
	E33QE41Q^*^ Spm	0.74 ± 0.10	0.22 ± 0.01	3.0 × 10^2^	1
	E33QE41Q^*, #^ Spd	4.87 ± 0.46	0.03 ± 0.01	6.2 × 10^0^	1
Acceptor site residues	E72Q Spm^*^	0.07 ± 0.01	0.04 ± 0.01	5.7 × 10^2^	1
	E72Q Spd^*^	1.57 ± 0.08	0.45 ± 0.01	2.9 × 10^2^	1
	E75Q Spm	0.32 ± 0.01	22.4 ± 0.2	7.0 × 10^4^	1.5 ± 0.1
	E75Q^#^ Spd	4.37 ± 0.20	17.8 ± 0.5	4.1 × 10^3^	1.4 ± 0.1
	E84Q Spm	0.21 ± 0.01	3.2 ± 0.1	1.5 × 10^4^	1.6 ± 0.2
	E84Q Spd	1.21 ± 0.03	4.9 ± 0.1	4.0 × 10^3^	1.9 ± 0.1
	Q86A Spm	0.34 ± 0.01	17.6 ± 0.2	5.2 × 10^4^	2.0 ± 0.2
	Q86A Spd	1.17 ± 0.05	10.0 ± 0.2	8.5 × 10^3^	1.5 ± 0.1

* Fitted with Michaelis–Menten equation; all other data were fitted with the Hill equation.

# Did not reach complete saturation even at 10 mM.

#### Note about system complexity and interpretation of kinetic results

It is important to note that in this system the allosteric effector and the acceptor substrate are chemically identical molecules. Therefore, the experimental design of titrating substrate concentration for a kinetic evaluation of enzymatic turnover and apparent affinity includes two molecular binding events: binding in the allosteric site and binding in the acceptor site. An observed kinetic response curve is also influenced by many potential iterative binding events: homotropic cooperativity between allosteric sites, homotropic cooperativity between active sites, and/or heterotropic allosteric coupling between active and allosteric sites. It follows that any single substitution in either site has the potential of altering ligand affinity in the allosteric site, ligand affinity in the acceptor site, homotropic cooperativity between allosteric sites, homotropic cooperativity between acceptor sites, heterotropic allosteric coupling between acceptor and allosteric sites, or any combination of these properties. This intrinsic complexity of possible outcomes precludes us from *a priori* speculating exact outcomes for the substitutions created in the present study. Nevertheless, our goal was to lay a foundation for querying their functional roles while acknowledging that limitations exist regarding interpretations due to the complexity of the system.

#### Apparent Affinities for Spm and Spd (K_m_ or S*_0.5_*)

We found that all substituted enzymes showed a similar trend to the WT enzyme by exhibiting a higher apparent affinity (*S*_0.5_) for Spm compared with Spd (Table 2 and Figure 4B,C). Only two significant differences to this trend were observed: E37Q (in the allosteric site) and Q86A (in the acceptor site) showed a slight improvement in apparent affinity compared with WT for both Spm and Spd (E37Q: ∼1.4- and ∼2.5-fold, respectively, and Q86A: ∼1.3-fold for both) (Table 2 and Figure 4B,C). However, the degree to which the apparent affinity between Spm and Spd substrates was altered for all substitutions varied. For example, three of the five substitutions in the allosteric site exhibited a significantly higher apparent affinity for Spm compared with Spd (E33Q (∼10-fold), E34Q (∼6-fold), and E41Q (∼5-fold), whereas E37Q did not exhibit as dramatic of a difference (∼3-fold) (Table 2 and Figure 4B,C). The same overall trend was observed when substitutions were made in the acceptor site, with three of the four substitutions exhibiting a significantly higher apparent affinity for Spm compared with Spd (E72Q (∼22-fold), E75Q (∼14-fold), and E84Q (∼6-fold); Q86A did not exhibit as dramatic of a difference (∼3-fold) (Table 2 and Figure 4B,C).

As noted above, in the WT 4MHD crystal structure of VcSpeG in complex with Spd, E33 and E37 in the allosteric site interact directly with the *N*^1^ terminal amine of Spd. Our kinetic results show that E33Q more significantly impairs the enzyme’s apparent affinity for Spd compared with E37Q and all other residue substitutions in the allosteric site, noting its criticality for binding Spd at this site. E33Q maintains a similar apparent affinity for Spm compared with WT despite E33 forming direct interactions with the *N*^1^ terminal amine of Spm in the allosteric site in the WT 6E1X crystal structure. On the other hand, E37Q improves the apparent affinity of the enzyme for both Spm and Spd, with a larger improvement toward Spd. Thus, this residue is likely critical for allosteric effector binding and specificity. In the acceptor site, E84 and Q86 form direct or water-mediated side chain interactions with the *N*^1^-AcSpm product, whereas E72 and E75 do not. Instead, E72 and E75 are mediators of ordered waters that may be necessary to maintain locations of specific orientations of residues in the active site. Despite their lack of direct side chain interactions, both of these substitutions (E72Q and E75Q) significantly impair the enzyme’s apparent affinity for Spd while maintaining similar apparent affinities for Spm compared with WT. Thus, their acidity is critical for Spd binding in the acceptor site. Similar to E37Q in the allosteric site, Q86A improved the apparent affinities for both substrates compared with WT, further indicating a possible role for this residue in substrate binding and specificity.

#### Enzymatic turnover toward Spm and Spd (k_cat_)

We found that the turnover number (*k*_cat_) for the WT enzyme was similar for both Spm and Spd substrates (∼1.1-fold). However, this trend was inconsistent across substitutions in both allosteric and acceptor sites. For example, the E34Q and E41Q substitutions in the allosteric site showed ∼1.7- and ∼1.4-fold higher turnover for Spm compared with Spd, whereas the E33Q and E37Q substitutions in the allosteric site showed the opposite effect with ∼2.1- and ∼1.5-fold higher turnover for Spd compared with Spm (Table 2 and Figure 4B,C). Similar trends were observed for the substitutions in the acceptor site, where E75Q and Q86A had an ∼1.3- and ∼1.8-fold increase in turnover for Spm compared with Spd. In contrast, E72Q and E84Q substitutions in the acceptor site had ∼11.2- and ∼1.5-fold increases in turnover for Spd compared with Spm (Table 2 and Figure 4B,C).

When we examined which substitutions at each site caused the greatest decrease in turnover for both polyamines compared with WT, we found the following. E34Q exhibited significantly impaired turnover for both Spm and Spd compared with WT, making it the least active of all substitutions in the allosteric site (Table 2 and Figure 4B,C). Similarly, E72Q exhibited the most significantly impaired turnover for both polyamines compared with WT and all substitutions tested across both allosteric and acceptor sites. While not as significant as E72Q, the E84Q substitution in the acceptor site showed a substantial decrease in turnover for both Spm and Spd compared with WT. Thus, these three substitutions had the most pronounced effect on turnover toward both substrates and implicates these residues as being critical for activity.

Several of the most notable changes in turnover due to substitution probing might best be evaluated by considering interactive networks within the structure. The rationale for why the E34Q residue in the allosteric site could cause such a drastic decrease in turnover likely stems from the fact that E34 forms significant contacts with residues in the adjacent protomer. It appears these contacts would be reduced, and the allosteric loop that harbors additional acidic residues (E33, E37, and E41) for polyamine binding to the allosteric site may become destabilized when this residue is altered. Another example where a significant decrease in turnover is observed is for the E72Q residue in the acceptor site. This residue does not directly interact with the polyamine in the acceptor site. Instead, E72 forms several water-mediated interactions with the *N*^1^-terminal amine of Spm and other residues in the allosteric site as well as side chain interactions with Q86 and water-mediated interactions in the acceptor site. Disrupting these interactions of the E72 residue is thus detrimental for turnover and indicates it may play a networking role between the two polyamine-binding sites of the enzyme. Finally, the E84 residue forms several water-mediated interactions with multiple amines of *N*^1^-AcSpm in the acceptor site. The primary effect of the E84Q substitution is on the enzyme’s ability to turnover substrate, and is likely due to a somewhat lessened stabilization of the polyamine in the acceptor site. These networking interactions may be key considerations for how the allosteric and active sites function.

#### Catalytic Efficiencies for Spm compared with Spd (k_cat_/K_m_ or k_cat_/S_0.5_)

When we compared the catalytic efficiencies (*k*_cat_/*S*_0.5_) of different substitutions toward both Spm and Spd compared with WT, we found that E37Q and E41Q in the allosteric site were both more efficient toward Spm, whereas only E37Q was more efficient toward Spd (Table 2 and Figure 4B,C). On the other hand, E33Q and E34Q had lower catalytic efficiencies for both Spm and Spd compared with WT. Therefore, maintaining polarity in the allosteric site improved catalytic efficiency toward the longer chain polyamine Spm for E37Q and E41Q, but not E33Q or E34Q. Additionally, while all substituted enzymes exhibited decreased catalytic efficiencies for Spd compared with Spm, the most efficient enzyme toward Spd was E37Q, and it was more efficient than WT for this substrate. This indicates a substitution at this position in the allosteric site can tune substrate specificity toward shorter polyamines like Spd and reach a catalytic efficiency that is the same order of magnitude for longer-chain polyamines like Spm. Overall, these results show that shifting these selected residues from acidic to polar does not severely affect trends in apparent affinity for Spm, but it can more dramatically affect the apparent affinity and turnover of some mutants for Spd.

Similar to outcomes of substitutions in the allosteric site, substitutions in the acceptor site also improved catalytic efficiency toward the longer-chain polyamine Spm. For example, E75Q and Q86A were more catalytically efficient toward Spm compared with WT (Table 2 and Figure 4B,C). Interestingly, E75Q not only improved the catalytic efficiency of the enzyme toward Spm, but it was also the most active (*k*_cat_) and efficient (*k*_cat_/*S*_0.5_) enzyme toward Spm. Thus, it appears that this residue is key for improving substrate specificity in the acceptor site toward longer chain polyamines. The Q86A mutant also showed an improved catalytic efficiency toward Spm, albeit lower than E75Q, indicating that substitutions at this position are allowable. Overall, these results show that these substitutions do not severely affect trends in apparent affinity for Spm, but they can more dramatically affect the apparent affinity and turnover of some mutants for Spd. When considering the allosteric site and acceptor site substitutions together, we also found that the most substantial decreases in catalytic efficiencies for Spm were caused by E72Q, E34Q, E84Q, and E33Q (ranked by least efficient to most efficient) and are driven by decreased turnover (E72Q and E34Q) or decreased apparent affinity (E84Q and E33Q) as mentioned above. Thus, the kinetic effects of altering these residues are widespread in both allosteric and acceptor sites of the protein and not dominated in one site over the other.

#### Comparison of apparent cooperativity (n*_H_*) when Spm and Spd are substrates

As introduced earlier, it is important to bear in mind that the observed cooperative responses in kinetic profiles can arise from homotropic interactions among the 12 acceptor sites, homotropic interactions among the 12 allosteric sites, and heterotropic interactions among the acceptor sites and allosteric sites. Any change in an observed cooperativity is a composite of the influence of substitutions on all of those contributing interactions. When we compared single substitutions in the allosteric site to WT, we found the majority of the substitutions maintained or only moderately modified positive cooperativity when Spm was the substrate. Similar results were observed for most substituted enzymes when Spd was the substrate. However, the exceptions were that the E33Q and E41Q enzymes showed significantly increased positive cooperativity when Spd was the substrate (i.e., increased cooperativity when comparing E33Q and E41Q to WT when Spd is the substrate and increased cooperativity for these two substituted proteins when comparing Spd to Spm as the substrate).

None of the substitutions in the acceptor site caused the highest level of increase in cooperativity found in the set of substitutions made in the allosteric site. E72Q was the only single substitution that caused the enzymes to exhibit hyperbolic behavior (i.e*.*, reduced positive cooperativity) when both Spm and Spd were used as substrates. We interpret this result to mean that either communication between the allosteric and active sites is disrupted or the allosteric effector is unable to bind when E72 is substituted. Due to the large number of conserved acidic residues in the allosteric site that are still present and potentially able to bind effectors when this residue (E72Q) is substituted, we hypothesize that the former explanation is more plausible. However, additional experiments will be required to distinguish these options. Thus, while several substitutions exhibit slight alterations in positive cooperativity compared with WT for both Spm and Spd, the only single substituted protein that exhibits hyperbolic behavior is the E72Q enzyme. This indicates that E72 is critical for the allosteric network of this enzyme and provides a significant insight as to how these two sites are working in concert.

#### Effect of substituting proposed catalytic residue in the donor site

Finally, we examined the Y134A mutant toward Spm and Spd and found a large loss of enzymatic activity (159-fold and 225-fold decrease in activity, respectively, compared with WT; Supplementary Figure S5). This outcome is consistent with our previous data that showed the SpeG enzyme from *E. coli* utilized the equivalent residue as a general acid for catalysis [32] and confirms this conserved residue is critical across species.

### Substituting both E33 or E41 in the allosteric site significantly impairs enzyme activity but single residue substitutions can compensate for each other

**Figure 5:**
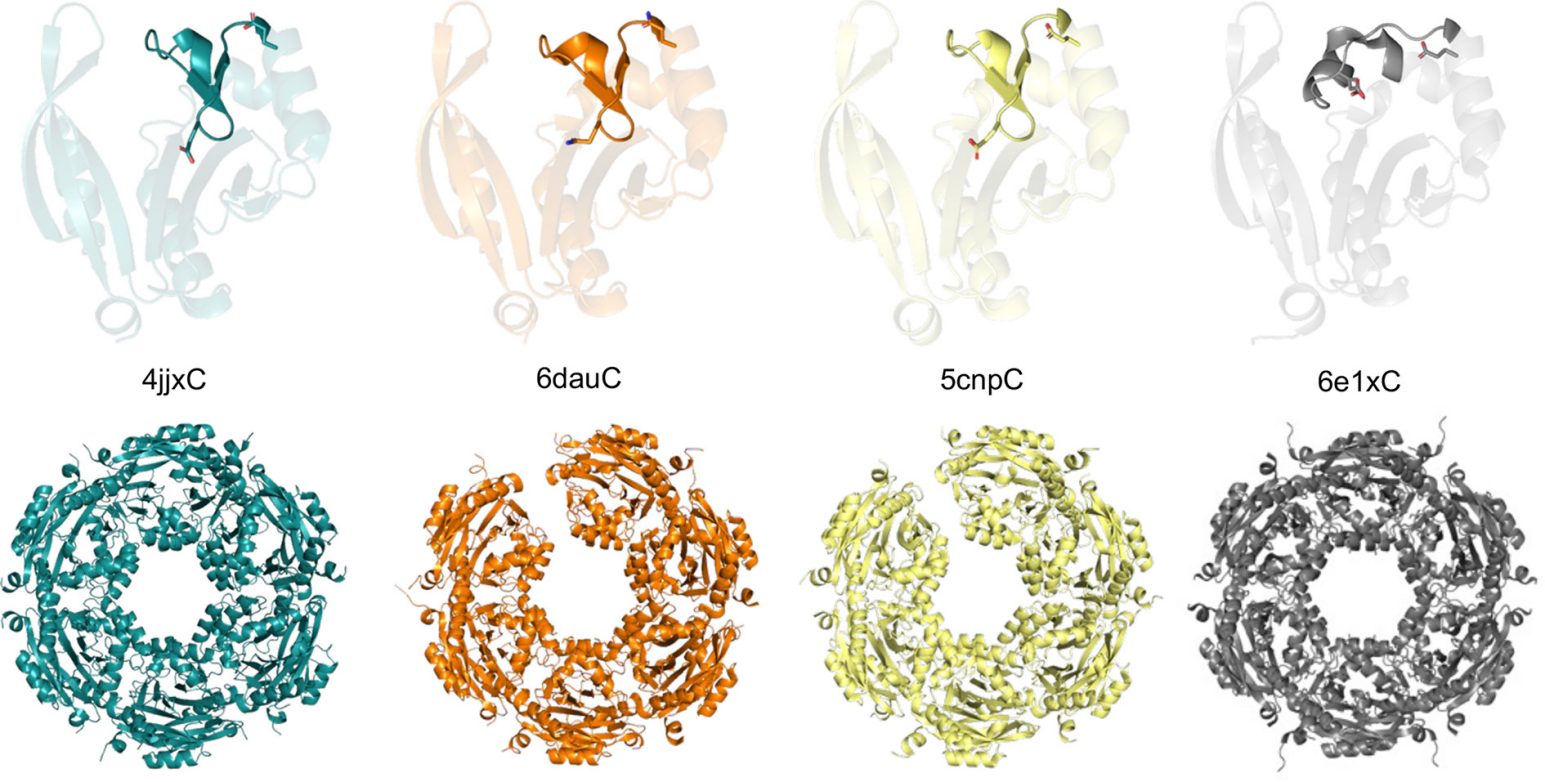
Comparison of the VcSpeG WT and E33QE41Q protein crystal structures (**Top**) A single protomer (chain C) of various VcSpeG crystal structures is shown in teal (WT; PDB ID: 4JJX), orange (E33QE41Q; PDB ID: 4JJX), yellow (WT; PDB ID: 5CNP), and gray (WT in complex with Spm and N^1^-AcSpm; PDB ID: 6E1X). E33(Q) and E41(Q) residues are shown with sticks and appear on the darker-colored allosteric loop. (**Bottom**) Corresponding dodecamers of 4JJX, 6DAU, 5CNP, and 6E1X structures. The 4JJX and 6E1X structures adopt closed conformations of the dodecamer, and the 6DAU and 5CNP structures have open conformations of the dodecamer.

One of the challenges in studying the SpeG enzyme is the difficulty in designing studies that report on isolated functions in the allosteric and acceptor sites because the substrate and allosteric effector are identical molecules. In an attempt to remove ligand binding to the allosteric site and thus isolate the acceptor site function, we sought to generate a substituted enzyme that would directly relieve polyamine binding in the allosteric site. Such a substitution is expected to remove the allosteric signal, while still enabling turnover in the active site. Since none of the acidic residues in the allosteric site that we substituted showed significantly decreased activity, we suspected that there was redundancy among these residues towards binding polyamine to this site. Therefore, we generated the E33QE41Q doubly substituted protein, tested its enzymatic activity, and determined its crystal structure since both E33 and E41 form direct interactions with Spm in the WT 6E1X structure (Table 1 and Figure 5). We found that the E33QE41Q enzyme activity (*k*_cat_) decreased to near baseline when using either of the two substrates (57-fold and 450-fold decrease for Spm and Spd, respectively, compared with WT). Cooperativity (n_H_) was also absent for both substrates (Table 2 and Figure 4B,C), indicating that the allosteric signal is disrupted compared with other single substituted enzymes in the allosteric site. This hyperbolic behavior and relief of positive cooperativity for the E33QE41Q enzyme mirrors what we observed with the E72Q mutant. However, in this case (E33QE41Q), we hypothesize the reduced cooperativity is likely due to lack of allosteric effector binding in the allosteric site, which is supported by the crystal structure (discussed below). The two types of substitutions in each site (E33QE41Q-allosteric and E72Q-acceptor) are key to understanding the complex SpeG allosteric system.

Despite performing crystallization trials of the doubly substituted protein in the presence of 5 mM Spm, the E33QE41Q protein structure (PDB ID: 6DAU) did not bind Spm in the allosteric site and instead adopted a conformational state that mimicked the apo form of the protein (Figure 5). Since the WT enzyme 6E1X structure showed that smaller amounts of contaminant Spm were able to bind with high affinity at the allosteric site, this means the Spm binding affinity for the double mutant is severely impaired and may explain the significantly reduced turnover. Additionally, we found that the E33QE41Q structure adopted an open conformation of the dodecamer, similar to what we observed previously for several other VcSpeG structures (PDB IDs: 5CNP and 7KWH; Figure 5) [11,18]. Thus, both E33 and E41 residues are key for high affinity polyamine binding in the allosteric site and homotropic allosteric behavior of the enzyme.

## Discussion

### The SpeG allosteric site binds non-acetylated spermine and there are two distinct polyamine binding sites in this protein

The presence of Spm in the allosteric site of the crystal structure was unexpected since only *N*^1^-AcSpm was used for co-crystallization and soaking, not Spm. We suspect the non-acetylated Spm was a contaminant from the commercially supplied *N*^1^-AcSpm (purity ≥97%). Even high-purity standards can contain small amounts of structurally similar species that are not always completely resolved or quantified analytically by suppliers. Based on the well-defined electron density for non-acetylated Spm in the allosteric site in chains A–D, and despite its presence as a low-level contaminant, the structure suggests the allosteric site binds Spm instead of *N*^1^-AcSpm. The partial disorder of the Spm ligand observed in the allosteric site of chains E and F correlates with conformational heterogeneity of the adjacent allosteric loop in these protomers (Supplementary Figure S4). Although the loop is well-defined in the electron density, it adopts multiple conformations, which likely modulate stabilizing interactions with the ligand, leading to partial occupancy and/or disorder of the terminal polyamine moiety. Since Spm was not intentionally included during crystallization, its presence most likely arises from trace contamination in the ligand preparation; its low effective concentration may further contribute to incomplete occupancy at certain allosteric sites. Our previous study proposed the allosteric site of the VcSpeG enzyme had a higher binding affinity (low micromolar range) for Spd/Spm compared with the acceptor site (high micromolar range). This was based on ITC and crystallization studies [11]. The presence of non-acetylated Spm in the allosteric site and *N*^1^-AcSpm in the acceptor site in the 6E1X crystal structure is consistent with our previously proposed kinetic mechanism [11] and contributes to our understanding of the selectivity of the different sites for polyamines. It also refutes a previously proposed *E. coli* SpeG mechanism where Spd in the allosteric site approaches the active site using a water network [27]. In this present study, we did not observe any intermediary polyamine binding site between the allosteric and acceptor sites that would support this previously proposed mechanism. Therefore, we maintain that separate polyamine binding sites, one for the polyamine that binds as an allosteric effector and one for the polyamine that becomes acetylated, are distinct and are a hallmark of this novel homotropic allosteric GNAT enzyme.

### Hypothetical model for roles of the conserved acidic and polar residues in SpeG proteins and their influence on cross-talk between allosteric and acceptor sites and polyamine selectivity

**Figure 6:**
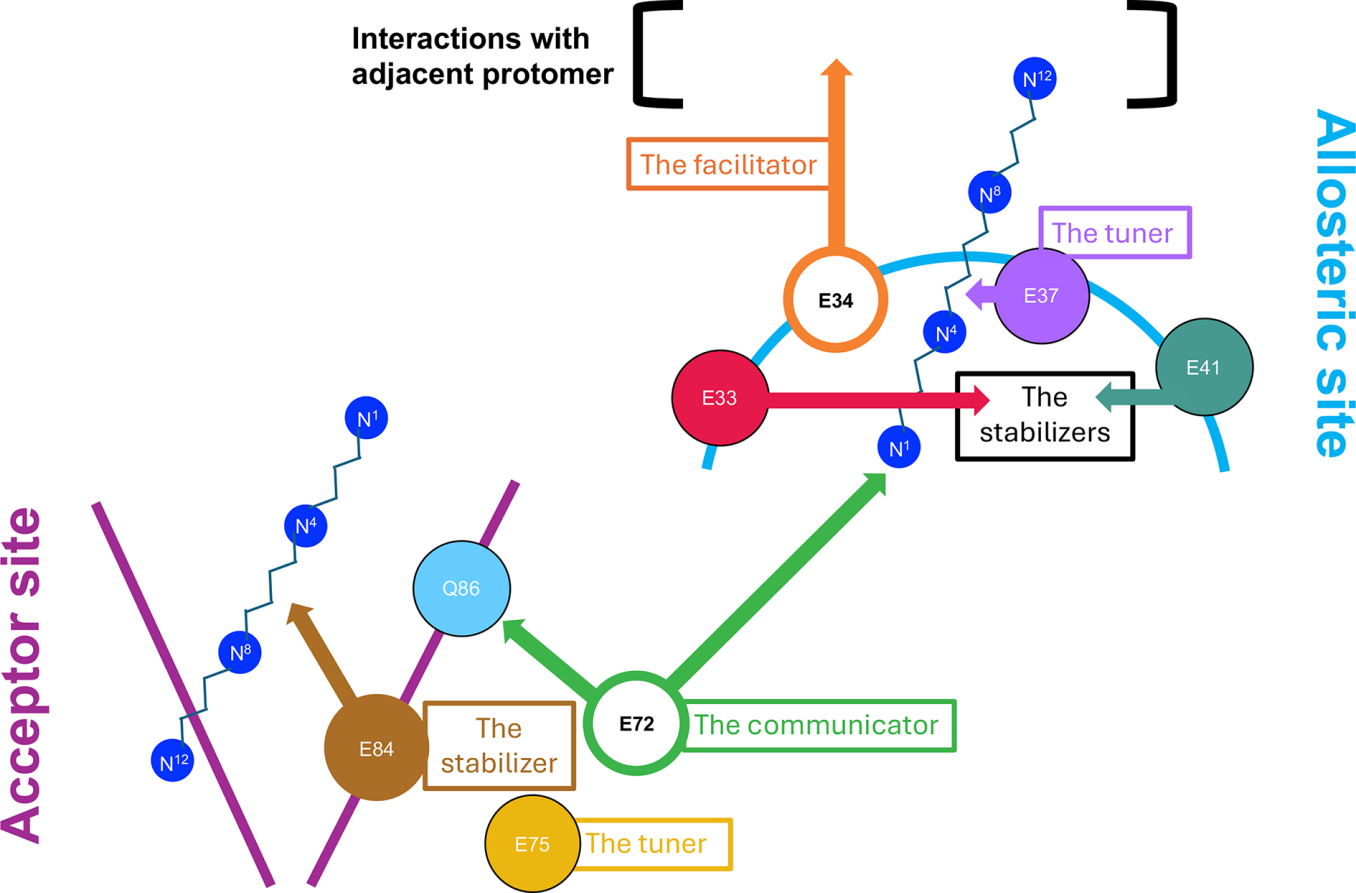
Hypothetical model of proposed roles of conserved acidic residues in the allosteric and acceptor sites of SpeG proteins

In the present study, we substituted conserved acidic residues in the allosteric and acceptor sites of bacterial SpeG homologs in the VcSpeG protein to determine their importance on kinetic activity and substrate and allosteric effector specificity. Collectively, we found that there were several key residues in both the allosteric and acceptor sites that were critical for activity, while others may be implicated in either providing supporting roles or aiding polyamine selectivity in these sites. Based on both the kinetic and structural data, we propose that the two sites are linked through an intricate series of interactions between residues that bind polyamines at both allosteric and acceptor sites and a network of water molecules (Figure 4A). Here, we present a hypothetical model (Figure 6) that puts these results in context while recognizing the limitations to data interpretation mentioned above for this complex allosteric enzyme.

First, in the allosteric site, the substitution that had the most significant effect on reducing activity was E34Q. We have termed this residue the ‘*allosteric site facilitator’* in our hypothetical model since it seems to stabilize the allosteric loop for polyamine binding and facilitates intersubunit interactions rather than directly interacting with polyamines in the allosteric site (Figure 6). If this loop is not stabilized, we hypothesize that the other conserved glutamate residues, such as E33 and E41, are unable to form proper contacts with polyamines in this site, and therefore binding would be significantly impaired. Our results showed that each of these residues (E33 and E41) can be singly substituted, but substitutions to both residues (E33Q and E41Q) cause a drastic reduction in turnover (*k*_cat_) and catalytic efficiency (*k*_cat_/*K*_m_). We also observed these doubly substituted enzymes showed a relief of cooperativity, unlike their singly substituted counterparts. Thus, single substitutions are intrinsically redundant to each other in these positions, but alterations to both residues, or too many disturbances in this site, result in a more severely impaired enzyme where cooperativity is also relieved. Since these residues interact directly with polyamine at the allosteric site, we have termed these residues the ‘*allosteric site polyamine stabilizers’* in our hypothetical model (Figure 6). We also hypothesize that in addition to E34 facilitating allosteric loop stabilization, the E33 and E41 residues must remain acidic to enable polyamine binding and transition of the allosteric loop to a more ordered alpha helix, as we observed for the 6DAU (E33QE41Q) crystal structure compared with WT. Finally, since the E37Q substitution in the allosteric site improved catalytic efficiency toward both Spm and Spd, in our hypothetical model we have termed this residue the ‘*allosteric site tuner’* (Figure 6). This residue does not interact with polyamines directly, but we hypothesize that altering the acidity at this position can tune the site for specific polyamines by slight adjustments to water-mediated interactions with the polyamine in the allosteric site and other water-mediated interactions with residues of the acceptor site, such as E72 (Figure 4A).

In the acceptor site, we found that the E72Q substitution exhibited the most drastic reduction in activity of all substituted residues in the acceptor site despite having no direct interactions with polyamines in either site. This significant reduction in turnover, combined with significantly reduced cooperativity, implicates E72 in facilitating communication between allosteric and acceptor sites. Thus, we have termed it the ‘*acceptor site communicator’* in our hypothetical model (Figure 6). Since the E84 residue directly interacts with the central portion of the polyamine in the acceptor site and several kinetic parameters compared with WT were reduced when this residue was substituted, we have termed this residue the ‘*acceptor site stabilizer’* in our hypothetical model (Figure 6). Similar to the E37 residue in the allosteric site, the E75 residue does not directly interact with polyamine in the acceptor site. However, reducing the acidity of this residue (E75Q) improves the turnover for both Spm and Spd compared with WT and significantly improves catalytic efficiency toward Spm compared with WT. Therefore, we have termed the E75 residue the ‘*acceptor site tuner’* in our hypothetical model (Figure 6). The effect of the Q86A substitution on kinetic parameters compared with WT was relatively unchanged compared with other residues in this site. Thus, we did not propose a specific role for Q86 outside of its observed structural interactions with the polyamine in the acceptor site of the 6E1X WT crystal structure. Additionally, since this residue is not conserved across all SpeG enzymes, we hypothesize that there is flexibility in the identity of this residue at this site and it may be related to polyamine binding and stabilization rather than a direct kinetic effect.

Ultimately, we observed that removing the acidity of many residues in both allosteric and acceptor sites still enables polyamine binding and turnover, which indicates acidic amino acid redundancy in these sites enables some fluidity to how polyamines bind to these sites. However, the E34 residue in the allosteric site and E72 residue in the acceptor site are critical, and relatively minor substitutions at these positions cause severe alterations to enzyme turnover.

### Significance of allostery in the SpeG protein

Despite identifying key residues within each of the polyamine binding sites of the SpeG enzyme that were critical for activity and polyamine binding, several questions remain regarding the significance of having an SSAT enzyme that is allosteric. For instance, why would it be beneficial for this bacterial enzyme (SpeG) to be allosteric, while its eukaryotic counterparts and other types of bacterial SSAT enzymes, such as BltDs and PaiAs, are not? Since it is not yet known whether all types of SSAT enzymes are conserved across different bacterial species, it is possible that organisms whose genomes encode a functional SpeG enzyme require a more responsive control of polyamines compared with other species that may lack a SpeG homolog or that utilize a different type of SSAT enzyme. It is quite possible that different polyamine catabolic pathways and enzymes are required in different bacterial species [33], a prospect we are currently studying in our laboratory. In addition to responsiveness, our data hint at a potential purpose of allostery in the SpeG enzyme being related to altering substrate specificity. While the predominance of different types of polyamines in bacterial species can vary (e.g., Spd and putrescine are predominant polyamines in *E. coli*, but norspermidine is the predominant polyamine in *V. cholerae* [34]), the organism’s localization in diverse environments may expose these species to non-predominant types of polyamines that must be controlled. This occurs in environments such as the human gut microbiome [35] and plant rhizosphere [36]. Thus, it may be advantageous for species that are in diverse environments to have an allosterically regulated SSAT enzyme to respond. Furthermore, the large number of allosteric and active sites in the SpeG enzyme may be important in a species that encounters significantly variable levels of polyamines and already has other types of polyamine signaling mechanisms in place. Clearly, further study is required to gain a greater understanding of the significance of SpeG enzyme allostery, but the present study provides a strong starting point for further inquiry.

### Uniqueness of SpeG and its homotropic allosterism in the GNAT superfamily and outstanding questions that remain

To our knowledge, SpeG is the first GNAT enzyme that has been described to exhibit homotropic allosterism by using the same molecule as its allosteric effector and substrate. While unique in the context of what is currently known about homotropic allosteric GNAT enzymes, and due to the vastness of the GNAT superfamily, there is a high likelihood that other examples of this type of homotropic allosteric system may exist for other types of small molecules. Indeed, several examples of allosteric GNAT enzymes that utilize other protein domains or partners have been identified [37,38], so the concept of allosteric GNATs is not novel. However, an avenue for identifying allosteric GNATs is largely understudied and requires additional knowledge of GNAT functions—a concept that remains challenging. Despite this, there are still several outstanding questions that remain for the SpeG enzyme itself, especially around the observed homotropic allosterism *via* structural and kinetic studies.

One question is whether or not the allosteric effector could be one type of polyamine and a substrate be a different type of polyamine. Our prior studies with the *Staphylococcus aureus* SpeG enzyme showed that pre-incubating the enzyme with polyamine prior to enzymatic assays produced nearly equivalent catalytic efficiencies for both Spm and Spd [17]. However, we have observed that some SpeG enzymes can have different binding affinities for different polyamines at the allosteric site [11]. Therefore, this pre-incubation approach with one type of polyamine and then switching polyamine substrate to a different polyamine is likely not a viable strategy if there are known differences in polyamine binding affinities. Since we wanted to observe whether differences in kinetic parameters would occur for allosteric and acceptor site-substituted enzymes in the present study, we did not pre-incubate enzymes with polyamines prior to assaying. Thus, our observations are limited to single types of polyamines, i.e., the same polyamine as allosteric effector and acceptor substrate. Additionally, due to the limitations of our current assay, addressing this question will require a different type of approach whereby the allosteric effector would need to be “locked” within the allosteric site. Afterwards, kinetic assays could be performed to assess activity toward different polyamine substrates than what would be bound to allosteric sites of the protein.

Another outstanding question is whether or not each SpeG homolog is tuned to utilize the predominant polyamine of the specific organism or whether the enzyme is inherently capable of polyamine effector and substrate promiscuity. It could be advantageous for organisms that are present in diverse environments where they may encounter or import different polyamines, such as in the gut or soil microbiome, to have a promiscuous SpeG enzyme that can regulate intracellular concentrations of non-native polyamines. As mentioned above, the predominant polyamine in *V. cholerae* is norspermidine. However, our kinetic assays in the present study focused on less predominant polyamines in *V. cholerae.* Despite this, the VcSpeG enzyme efficiently acetylated Spm, which may indicate the latter possibility of SpeG enzymes being polyamine promiscuous (at least *in vitro*). Studies in our laboratory are currently ongoing to learn more about how the VcSpeG enzyme kinetic parameters for norspermidine compare to Spm and Spd, which will help address this unanswered question.

Finally, it is still unclear what the contributing factors are for the observed positive cooperativity of the enzyme. While much of our argument in the text is centered around the idea that the allosteric site has a higher affinity for polyamine than the active site and thus the positive cooperativity is related to binding effector, it is possible other interpretations are valid. The primary rationale for our argument for the allosteric site having a higher affinity for polyamine is based on our prior studies with the VcSpeG enzyme that showed high-affinity polyamine binding, crystal structures with the allosteric sites filled with polyamine molecules, and kinetic mechanistic studies that indicated the allosteric sites were likely filled first with polyamine prior to turnover in the active sites [11]. In the present study, we also present crystallographic data that show the allosteric site is filled with contaminant Spm from co-crystallization with *N*^1^-AcSpm rather than *N*^1^-AcSpm. Since there is currently no evidence for the reverse deacetylation reaction of *N*^1^-AcSpm by SpeG, it seems reasonable that Spm is indeed a contaminant species that binds with high affinity to the allosteric sites. Finally, when we performed substrate saturation curves for E72Q and E33QE41Q enzymes, we also observed that positive cooperativity was removed (i.e., curves were hyperbolic). While this loss of cooperativity could simply be due to removing cooperativity between substrate binding sites, it could also be due to removing coupling between allosteric and active sites of the protein. This hypothesis could be tested by molecular dynamics experiments in future studies. Ultimately, our study has laid the foundation for further biophysical, biochemical, and computational investigations, which are required to tease out the intricate details of the allosteric network of SpeG enzymes and validate or disprove our hypothetical model.

## Materials and methods

### Materials

All chemicals for enzyme assays and co-crystallization studies, including *N*^1^-acetylspermine, Spm (tetrahydrochloride), Spd (trihydrochloride), and AcCoA (trilithium salt), were purchased from Millipore Sigma. All other reagents were purchased at the highest quality available.

### Clones and site-directed mutagenesis

The WT VcSpeG clone in the ampicillin-resistant pMCSG7 vector (UniProtID: Q9KL03; cloning described previously [11]) was used to create the following single site substitutions: E33Q, E34Q, E37Q, E41Q, E72Q, E75Q, E84Q, Q86A, and Y134A. The doubly substituted enzyme E33QE41Q was created using the E33Q DNA as the template. Primers were designed using the NEBaseChanger tool (New England Biolabs) and were purchased from Integrated DNA Technologies. PCR reactions, ligation, and transformation of clones were performed using the Q5 Site-Directed Mutagenesis Kit (New England Biolabs), *E. coli* DH5alpha competent cells prepared using Mix & Go (Zymo Research), and procedures outlined as described before [18]. All substitutions were confirmed by DNA sequencing (Genewiz).

### Protein expression and purification for crystallization and enzyme kinetics studies

*Escherichia coli* BL21(DE3) cells were transformed with ampicillin-resistant clones, grown, expressed, harvested, and purified as described previously for crystallization [11] and for enzyme kinetics [18]. All proteins were frozen in aliquots (50–100 μl) and stored at −80°C for up to 1 month.

### Steady-state enzyme kinetics assays

All enzymatic assays were performed as described previously [18]. Kinetic assays were performed using proteins that were from a single freeze/thaw cycle. Additionally, each replicate assay was performed using a freshly thawed aliquot of protein. We have observed that SpeG proteins are less stable and lose activity after repeated freeze/thaw cycles (unpublished data). The kinetic reaction was performed discontinuously, where the enzyme reacts with substrates (AcCoA and polyamine) for 5 min at 37°C in the presence of 70 mM bicine (pH 8.0) and 20 mM NaCl. The total reaction volume was 50 μl. Next, the reaction was terminated with 50 μl of a solution that contains guanidine HCl to unfold the protein; this solution was comprised of 1 M guanidine HCl and 0.1 M Tris–HCl (pH 8.0). The product of the enzymatic reaction (CoA) is detected through its reaction with Ellman’s reagent (5,5'-dithio-bis-(2-nitrobenzoic acid) (DTNB)) in a 1:1 ratio and absorbance at A_415 nm_. This solution contained 0.2 mM DTNB, 0.1 M Tris–HCl (pH 8.0), and 1 mM EDTA; a total volume of 200 μl was added to the reaction and incubated for 10 min at RT before measuring the absorbance at A_415 nm_ with a filter-based BioTek ELX808 microplate UV/Visible spectrophotometer. The optimal reaction time for the discontinuous enzymatic assay was previously determined for the WT protein by monitoring the absorbance of the product at different reaction time points at saturating concentrations of substrates. The reaction time of 5 min produced an absorbance that did not saturate the detector and was within the linear range of the reaction as a function of time. The amount of enzyme that was used for all WT and mutant proteins was determined by varying protein concentration at constant concentrations of both substrates (0.5 mM AcCoA and 10 mM polyamine). For all WT and mutant proteins, the enzymes were diluted into a solution containing 50 mM bicine (pH 8.0) and 100 mM NaCl, and 10 μl of enzyme was used to initiate the reactions. The final amount of enzyme used for each polyamine substrate saturation curve ranged from 0.06 to 1.47 μM for Spm and from 0.06 to 2.71 μM for Spd across all WT and most mutant enzymes. Exceptions included E33QE41Q for Spm and Spd (4.5 and 9 μM, respectively), E72Q and Y134A for Spm (16 and 15 μM, respectively), and Y134A for Spd (15 μM). Polyamine substrate saturation curves were generated by holding AcCoA concentration constant at 0.5 mM and varying polyamine from 0 to 10 mM. No pre-incubation of enzyme with polyamine was performed prior to initiating the reactions. All data are the result of three to four biological replicate reactions, with a total of six to eight technical replicates for each enzyme. Enzyme activity in μmol min^−1^ mg^−1^ was determined by converting absorbance to nmol of product based on standards of l-cysteine with Ellman’s reagent. Since all proteins were pure, enzyme activity was then converted to s^−1^ using the molecular weight of the VcSpeG monomer (20.675 kDa).

### Protein crystallization

The sitting-drop vapor diffusion technique was used to set up crystallization of the WT and E33QE41Q VcSpeG proteins at 293 K. To obtain a complex of the WT protein with *N*^1^-AcSpm, the protein (8.7 mg/ml) was incubated with 20 mM *N*^1^-AcSpm on ice for 30 min prior to setting crystallization trials. Crystals of the WT VcSpeG protein in complex with *N*^1^-AcSpm were collected from a drop containing the protein-*N*^1^-AcSpm mixture, 5 mM magnesium chloride, 1% isopropanol, and 50 mM Tris–HCl (pH 8.5) buffer. The drop was equilibrated over a reservoir solution containing 10 mM magnesium chloride, 2% isopropanol, and 100 mM Tris–HCl (pH 8.5) buffer. Prior to setting up crystallization of the E33QE41Q protein, the protein (10 mg/ml) was incubated on ice with 5 mM Spm for 30 min. The crystallization drops were equilibrated over a reservoir solution containing 10 mM magnesium chloride, 10% isopropanol, and 100 mM Tris–HCl (pH 8.5) buffer and were incubated at 293 K. After 7 days, single crystals of both proteins were suitable for data collection. Crystals of the E33QE41Q protein were collected from a drop containing 5 mM magnesium chloride, 5% isopropanol, 5 mM Spm, and 50 mM Tris–HCl (pH 8.5) buffer. Crystals of the E33QE41Q protein were cryoprotected in 25% sucrose and then flash-frozen in liquid nitrogen. To obtain a complex with *N*^1^-AcSpm in the acceptor site, crystals of the WT protein were grown in the presence of *N*^1^-AcSpm and were also soaked in a drop containing 20 mM *N*^1^-AcSpm in the reservoir solution for 10 min prior to cryoprotection in 25% sucrose and freezing in liquid nitrogen.

### Protein structure determination and refinement

X-ray data sets for both structures were collected at Argonne National Laboratory (Argonne, IL) at the Life Sciences Collaborative Access Team (LS-CAT) beamline 21ID-F. The data were processed using HKL3000 [39], structures were solved using molecular replacement in Phaser [40] and were refined with REFMAC [41]. Previously determined VcSpeG protein structures PDB ID: 4MI4 and PDB ID: 5CNP were used as search models for the WT protein structure in complex with *N*^1^-AcSpm and E33QE41Q protein structure, respectively. Manual corrections of side chains, loops, ligands, and water molecules were performed in Coot [42,43], and the quality of structure models was validated by the Protein Data Bank (https://validate-rcsb-2.wwpdb.org) and MolProbity [44,45]. The WT VcSpeG protein in complex with *N*^1^-AcSpm was deposited using PDB ID: 6E1X; the VcSpeG E33QE41Q structure was deposited using the PDB ID: 6DAU. Refinement statistics and data collection parameters are shown in Table 1.

## Supplementary Material

Supplementary Figures S1-S5

## Data Availability

All supporting data are included within the main text of the article and supplementary materials. Additionally, all coordinates for crystal structures determined in the present study have been deposited into the Protein Data Bank with the accession codes PDB ID: 6E1X (VcSpeG WT protein in complex with Spm in the allosteric site and *N*^1^-AcSpm in the acceptor site) [46] and PDB ID: 6DAU (VcSpeG E33QE41Q protein) [47].

## Open Access

Open access for this article was enabled through an open access agreement between the Biochemical Society/Portland Press and the University of California.

## Abbreviations

AcCoA: acetyl coenzyme A
DTNB: 5,5'-dithio-bis-(2-nitrobenzoic acid)
GNATs: Gcn5-related *N*-acetyltransferases
MPD/MRD: (4S/4R)-2-methyl-2,4-pentanediol
*N*^1^-AcSpm: *N*^1^-acetylspermine
Spd: spermidine
Spm: spermine
SSATs: spermidine/spermine *N*-acetyltransferases
VcSpeG: SpeG enzyme from *Vibrio cholerae*
