# Supplementary material for: Roles of acidic residues in SpeG acetyltransferases—insights into importance for kinetic activity and polyamine binding in allosteric and acceptor sites
Source: Biochem J. 2026 Jun 26;483(6):1075–95. doi: 10.1042/BCJ20260162 (PMC13212360; doi:10.1042/BCJ20260162)
Supplement: Supplementary Figures S1-S5 [file BCJ-2026-0162_supp.pdf]

Supplemental Figures

Roles of acidic residues in SpeG acetyltransferases-insights into importance for kinetic activity and polyamine binding in allosteric and acceptor sites

Hazel N. Leiva Martel[1], Van Thi Bich Le[1], Ekaterina V. Filippova[2], Aron W. Fenton[3], Melissa Law[1], Martha Marquez-Ramirez[1], and Misty L. Kuhn[1]*

[1]Department of Chemistry & Biochemistry, San Francisco State University, San Francisco, California, USA

[2]Department of Biochemistry and Molecular Biology, Institute for Biophysical Dynamics, University of Chicago, Chicago, Illinois, USA

[3]Department of Biochemistry and Molecular Biology, The University of Kansas Medical Center, Kansas City, Kansas, USA

*Corresponding author: Misty L. Kuhn, Email: mkuhn@sfsu.edu

Spermine (allosteric site)

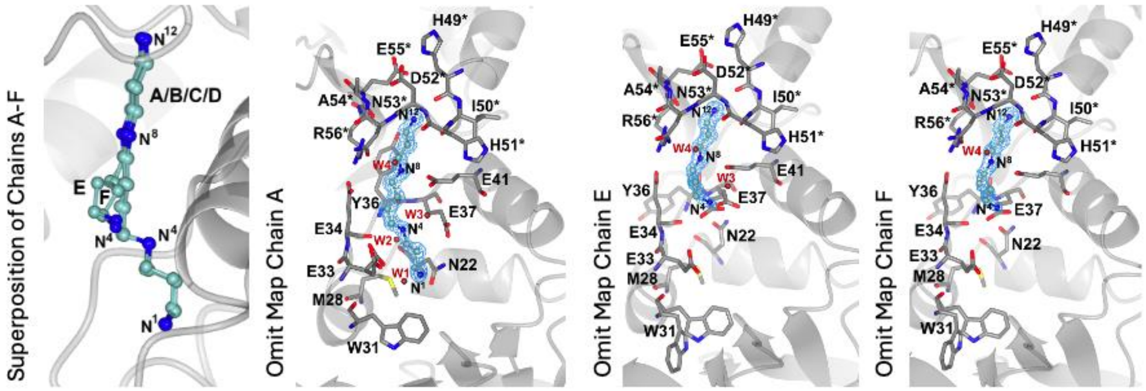

Supplemental Figure 1. Superposition and omit maps of spermine (Spm) within the allosteric site. Superposition of Spm in six protomers and electron density omit maps of Spm in protomers A, E, and F. Protein residues are shown in gray with non-carbon atoms colored in blue for nitrogen and red for oxygen. The Spm ligand is shown in light blue with non-carbon atoms (nitrogen) in dark blue. Water molecules are brown spheres. Residues from an adjacent protomer are denoted with an asterisk.

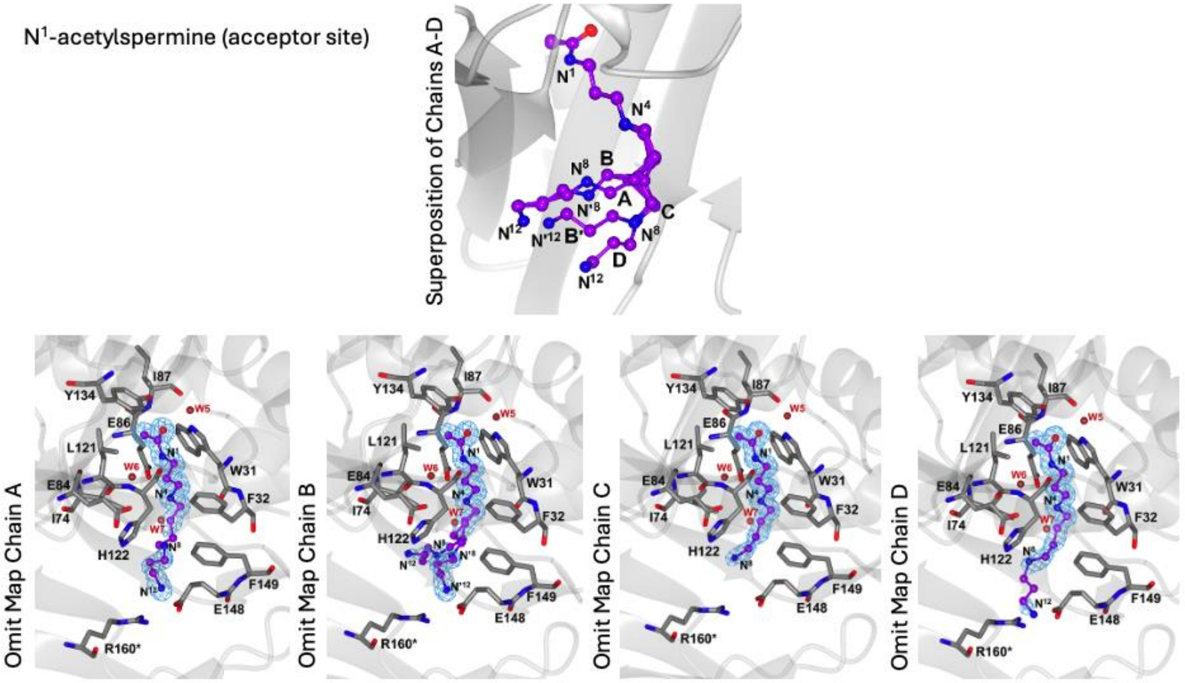

Supplemental Figure 2. Superposition and omit maps of N^1-acetylspermine (N^1-AcSpm) within the acceptor site. (Top) Superposition of N^1-AcSpm in four protomers (A-D). (Bottom) Electron density omit map of N^1-AcSpm in different protomers. Protein residues are shown in gray with non-carbon atoms colored in blue for nitrogen and red for oxygen. The N^1-AcSpm ligand is shown in purple with non-carbon atoms (nitrogen) in dark blue. Water molecules are shown with brown spheres. Atoms of N^1-AcSpm with partial occupancy are denoted with an appostrophe.

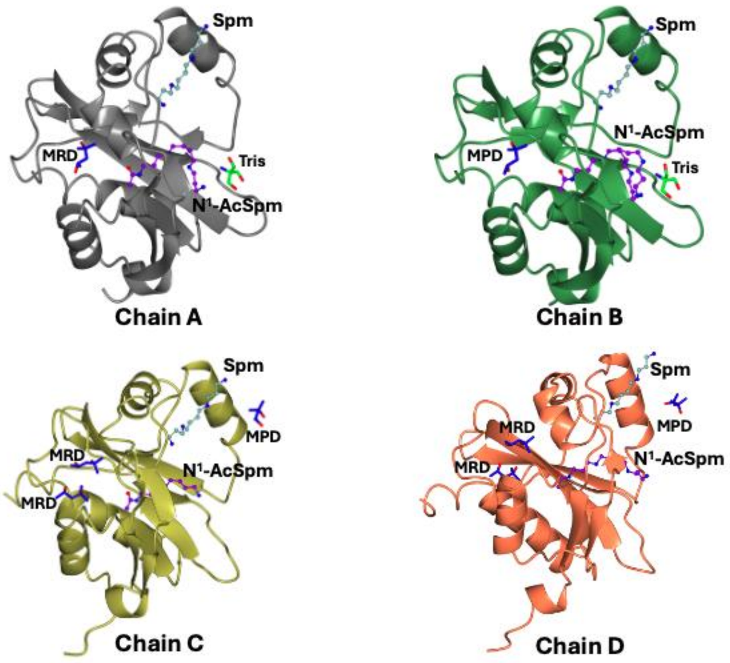

Supplemental Figure 3. Location of MPD/MRD and Tris buffer molecules relative to Spm and N^1-AcSpm in four chains of 6e1x VcSpeG crystal structure. Ligands are shown as sticks: N^1-AcSpm (purple), Spm (light blue), Tris (green), MPD/MRD (blue).

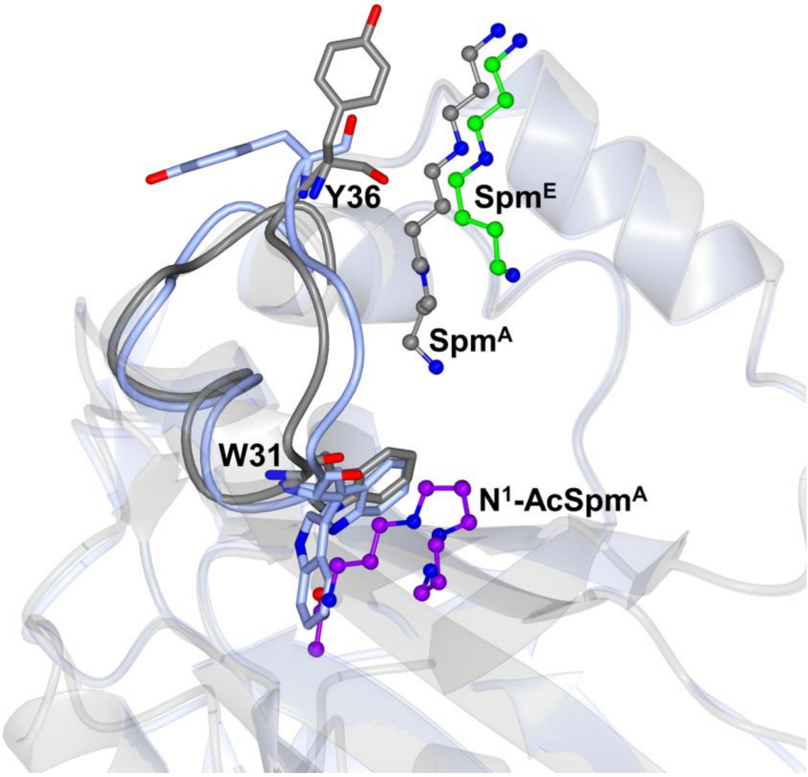

Supplemental Figure 4. Superposition of different protomers of VcSpeG in the 6e1x crystal structure. Superposition of chains A(B/C/D) (gray) and E(F) (light blue). Two conformations of W31 and Y36 are shown when Spm (from chain A; gray) is well-ordered and Spm (from chain E; green) partially disordered in the allosteric site; N^1-AcSpm in the acceptor site (chain A) is shown in purple. The conformation of W31 prevents binding of N^1-AcSpm in the acceptor site, while Y36 diminishes binding of Spm in the allosteric site.

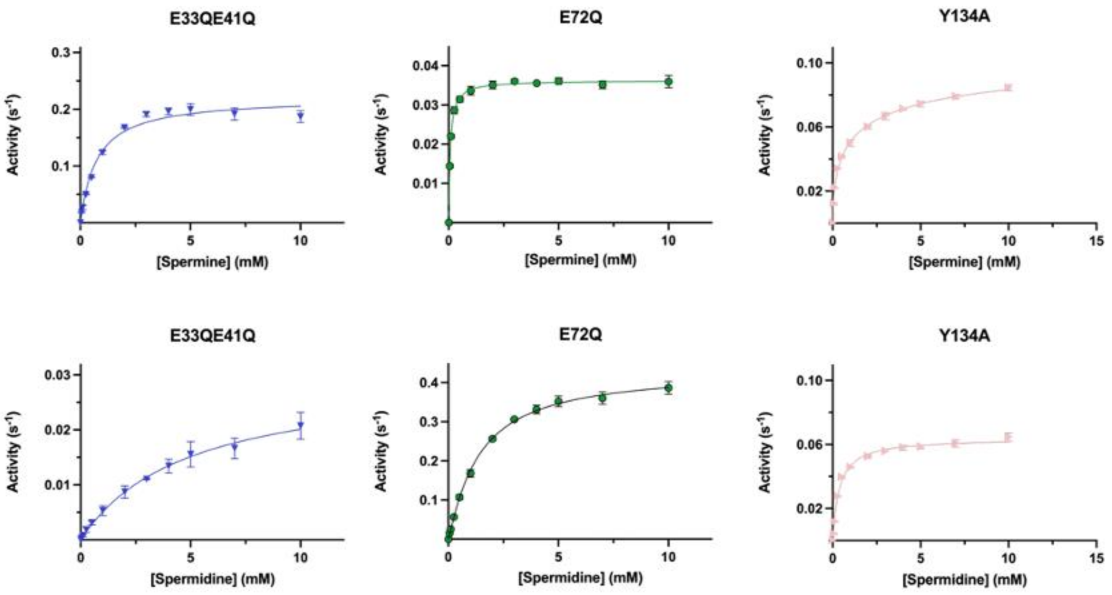

Supplemental Figure 5. Substrate saturation curves for VcSpeG substituted enzymes with low activity. These plots are a zoomed-in view of of the same data shown in **Figure 4B** plus Y134A enzyme activity toward both Spm and Spd.
